# Correction: Antimicrobial peptide-modified silver nanoparticles for enhancing the antibacterial efficacy

**DOI:** 10.1039/d4ra90040e

**Published:** 2024-04-29

**Authors:** Wenxi Li, Yongchun Li, Pengchao Sun, Nan Zhang, Yidan Zhao, Shangshang Qin, Yongxing Zhao

**Affiliations:** a School of Pharmaceutical Science, Zhengzhou University Zhengzhou Henan 450001 PR China zhaoyx@zzu.edu.cn +86 0371 67739546 +86037167739165; b Zhengzhou Traditional Chinese Hospital of Orthopaedics Zhengzhou Henan 450004 PR China; c Key Laboratory of Advanced Pharmaceutical Technology, Ministry of Education of China Zhengzhou Henan 450001 PR China; d Institute for Biological Interfaces 1, Karlsruhe Institute of Technology 76344 Eggenstein-Leopoldshafen Germany

## Abstract

Correction for ‘Antimicrobial peptide-modified silver nanoparticles for enhancing the antibacterial efficacy’ by Wenxi Li *et al.*, *RSC Adv.*, 2020, **10**, 38746–38754, https://doi.org/10.1039/D0RA05640E.

The authors regret that there was an error in the scale bars in Fig. 1E, 2C, and 3D in the original article.

Accordingly, Fig. 1E, 2C, and 3D in the original article should be replaced with the following revised Fig. 1E, 2C, and 3D. The conclusions of the paper have not been affected.
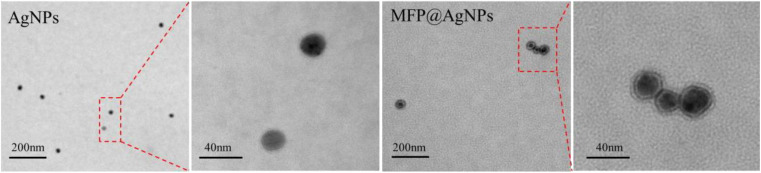


Fig. 1E. Representative TEM images of AgNPs and MFP@AgNPs-1.
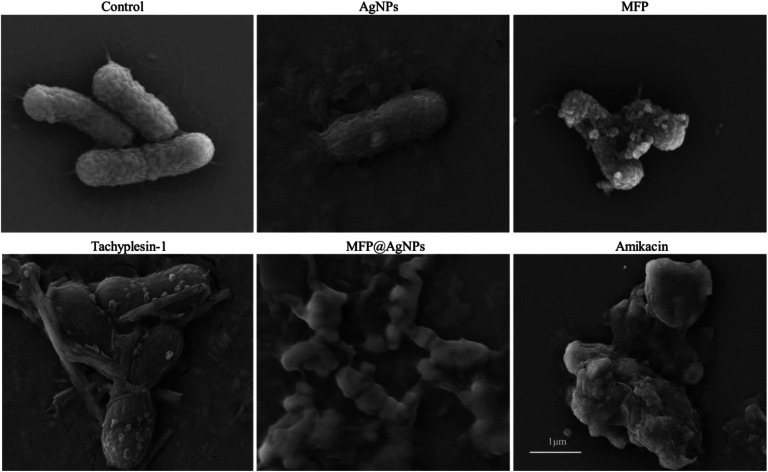


Fig. 2C. Morphology changes after treatment.
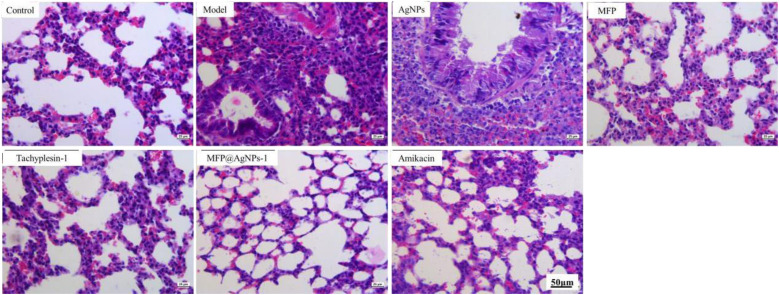


Fig. 3D. Representative images of histological analysis of the lung tissues.

The Royal Society of Chemistry apologises for these errors and any consequent inconvenience to authors and readers.

## Supplementary Material

